# Circulating metabolomic profile of the MIND diet and its relation to cognition in middle‐aged and older adults

**DOI:** 10.1002/imo2.61

**Published:** 2025-02-10

**Authors:** Hui Chen, Jie Shen, Yang Tao, Yaodan Zhang, Mengyan Gao, Yuan Ma, Yan Zheng, Geng Zong, Qing Lin, Lusha Tong, Changzheng Yuan

**Affiliations:** ^1^ School of Public Health, the Second Affiliated Hospital Zhejiang University School of Medicine Hangzhou China; ^2^ Cancer Epidemiology Unit, Nuffield Department of Population Health Oxford University Oxford UK; ^3^ Department of Epidemiology Harvard T. H. Chan School of Public Health Boston Massachusetts USA; ^4^ State Key Laboratory of Genetic Engineering, Human Phenome Institute, and School of Life Sciences Fudan University Shanghai China; ^5^ CAS Key Laboratory of Nutrition, Metabolism and Food Safety, Shanghai Institute of Nutrition and Health University of Chinese Academy of Sciences, Chinese Academy of Sciences Shanghai China; ^6^ Department of Neurology the First People's Hospital of Taizhou Taizhou China; ^7^ Department of Neurology, the Second Affiliated Hospital Zhejiang University School of Medicine Hangzhou China; ^8^ Department of Nutrition Harvard T. H. Chan School of Public Health Boston Massachusetts USA

## Abstract

The Mediterranean‐DASH Diet Intervention for Neurodegenerative Delay (MIND) diet has been related to a lower risk of dementia and better cognitive function. This study identified 47 circulating metabolites associated with the alternate MIND diet score (aMIND) in the UK Biobank (45,906 participants, 168 metabolites measured) and Whitehall II (6193 participants, 152 metabolites measured). Unsaturated fatty acids showed the strongest positive associations with the aMIND, and very low‐density lipoprotein measures and glycoprotein acetyls showed the strongest inverse associations. We constructed a metabolomic signature score (MIND‐MetS) to objectively reflect MIND diet adherence. Both aMIND and MIND‐MetS were associated with better cognitive outcomes in participants aged 55 years or older, and the MIND‐MetS partially mediated the aMIND‐cognition associations. In summary, the rapid and widely accessible nuclear magnetic resonance‐based metabolomic measure could objectively reflect MIND diet adherence. Moreover, our findings offered insights into the intricate connections among diet, metabolism, and cognition.

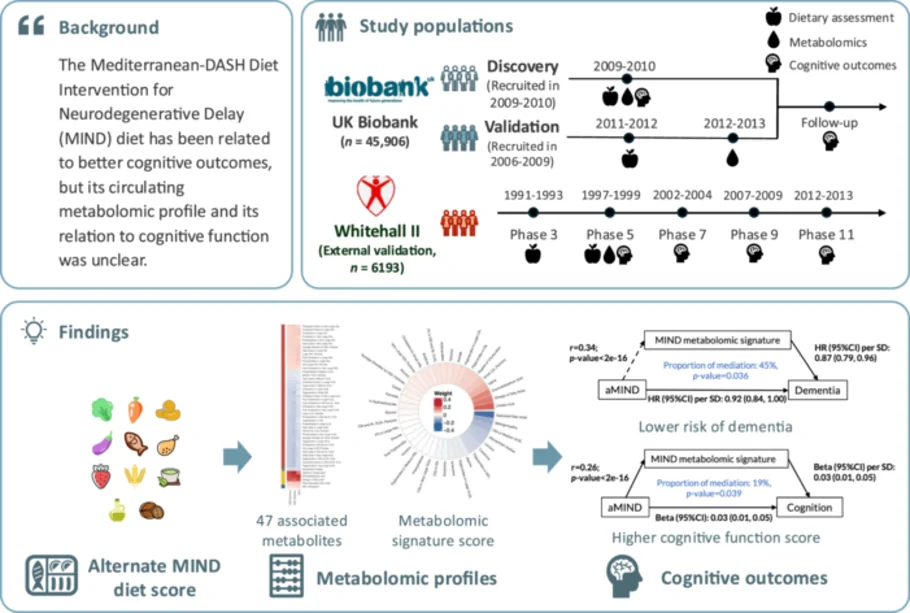

To the editor,

Dementia, characterized by severe cognitive and functional impairment, poses substantial burdens on healthcare systems and threatens the well‐being of older adults with limited treatments [1, 2]. Dietary factors have been associated with dementia [3, 4]. In particular, the Mediterranean‐Dietary Approaches to Stop Hypertension (DASH) Intervention for Neurodegenerative Delay (MIND) diet, which is a hybrid of the Mediterranean and DASH diets and uniquely encourages the consumption of antioxidant‐rich berries and green‐leafy vegetables [5], was associated with better cognitive function [6] and lower risk of dementia [7]. However, circulating metabolomic characteristics of the MIND diet and their roles in the associations between MIND and cognitive function remain unclear, which is important for identifying specific functional substances underlying the diet‐cognition associations. Metabolomic profiling provided valuable insights into the biological pathways underlying diet‐disease associations, such as the associations between the Mediterranean diet and cardiovascular diseases [8]. Comprehending which metabolites, as well as the extent to which degree they mirror adherence to the MIND diet, is of great importance for the discovery of novel intervention targets and the establishment of effective monitoring checkpoints. Given the antioxidant‐rich and anti‐inflammatory properties of the pivotal food groups (e.g., green‐leafy vegetables and berries) [3, 5], we hypothesized that the MIND diet‐induced favorable metabolomic profile may partially explain the MIND‐cognition associations.

In current study, we characterized the circulating metabolomic profiles of the MIND diet leveraging the dietary and circulating metabolomic data in two UK population‐based cohorts. Furthermore, we assessed the potential mediating role of the metabolomic signature of the MIND diet in the associations of the MIND diet with incident dementia and cognitive function.

## 1 RESULTS AND DISCUSSION

### Study overview and population

**FIGURE 1:**
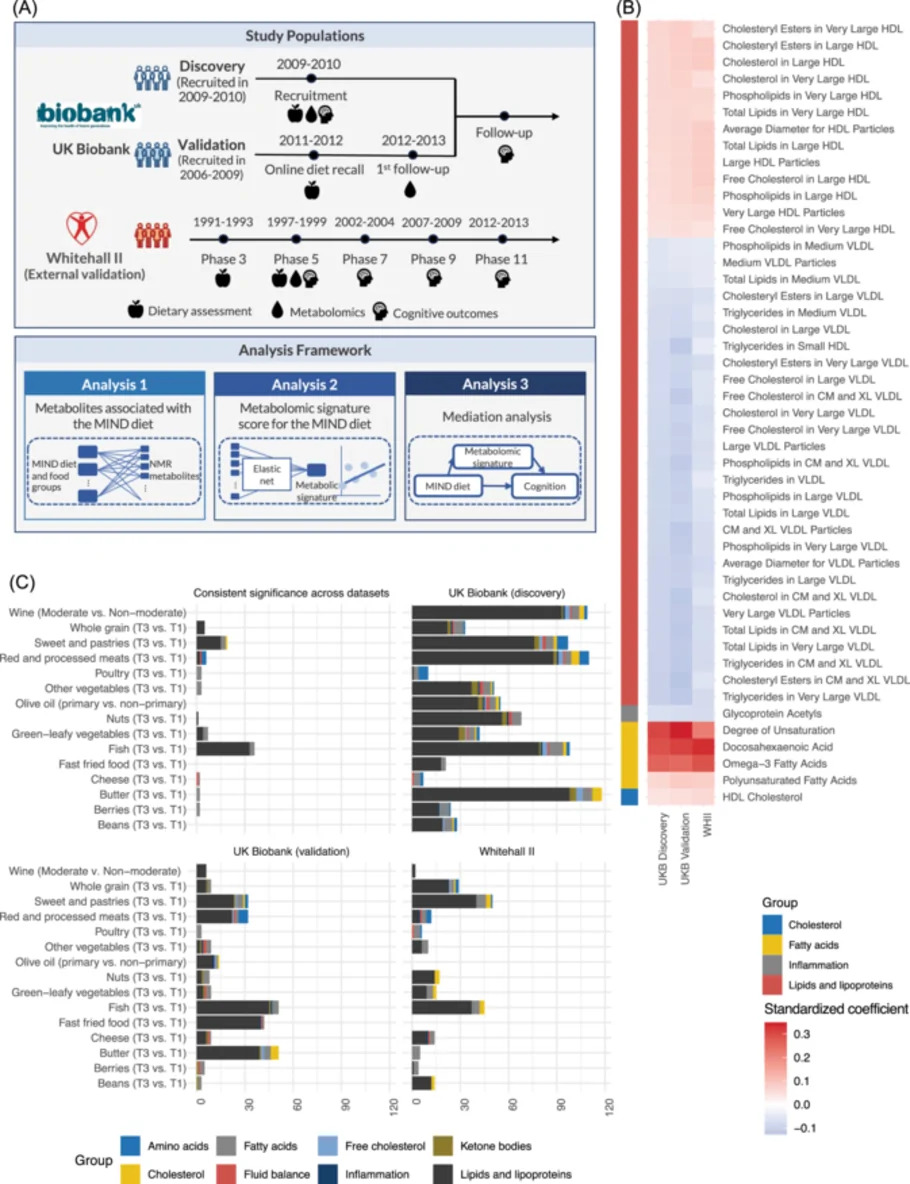
Study design and identification of metabolites associated with the MIND diet. (A) Study overview. (B) Metabolites consistently associated with the aMIND diet score (per three‐point increment) after adjustment for age, sex, total energy intake, ethnicity, marriage and partnership status, education level, physical activity, smoking, hypertension, diabetes, and cardiovascular diseases. (C) The number of metabolites associated with food groups in the MIND diet consistently in all three datasets and in each data set. aMIND, alternate MIND diet score; MIND, Mediterranean‐DASH Diet Intervention for Neurodegenerative Delay; NMR, nuclear magnetic resonance.

The overview of this study is shown in Figure 1A. This study is based on the UK Biobank (UKB, separated into a discovery cohort and an internal prospective validation cohort) and the Whitehall II study (WHII, serving as an external validation cohort). We included 45,906 UKB and 6193 WHII participants for analysis (Table S1). The UKB discovery cohort participants had a mean age (SD) of 56.4 (8.1) years at recruitment (2009–2010), and 54.9% were female. The UKB validation cohort participants had a mean age (SD) of 56.8 (7.4) years at recruitment (2006–2009), and 50.8% were female. The WHII participants had a mean age (SD) of 55.8 (6.2) years at baseline (1997–1999), and 29.0% were female. The missing rates of the metabolites are shown in Table S2.

### Metabolomic profiles of the MIND diet

Adherence to the MIND diet indicated by higher alternate MIND diet score (aMIND) was associated with 148 out of 168 (88%) metabolites in the UKB discovery cohort with a false discovery rate of <0.05 (Table S3), and 120 remained significant after Bonferroni corrections. Of the 148 associations, 83 (56%) were validated in the UKB internal validation cohort, and 47 (57%) were further replicated in the WHII cohort, including HDL‐Cholesterol, 41 lipid and lipoprotein subclass markers, four fatty acids, and one inflammatory marker (Figure 1B).

Higher aMIND was strongly associated with higher unsaturated fatty acid levels, such as omega‐3 fatty acids, lipoprotein particle sizes, ketone bodies, and fluid balance measures. For example, each three‐unit increment in the aMIND score was associated with 0.31, 0.29, and 0.26 higher z‐scores in the degree of unsaturation, docosahexaenoic acid, and omega‐3 fatty acids in the UKB discovery cohort. On the contrary, the strongest inverse associations were shown for very low‐density lipoprotein (VLDL)‐related measures, non‐polyunsaturated fatty acids, and inflammation‐related metabolites. For example, the coefficients for triglycerides in very large VLDL, average diameter for VLDL particles, and glycoprotein acetyls (mainly a1‐acid glycoprotein) were −0.10, −0.10, and −0.07, respectively. Among food groups, fish exhibited the most consistent associations with the metabolites (Figure 1C and Table S4). In previous studies, these metabolites are also associated with the Mediterranean diet [8] and the DASH diet [9], on which the MIND diet was based. In the UKB, unsaturated fatty acids and HDL measures have been associated with lower dementia risk, while LDL measures are associated with higher dementia risk [10]. In the Whitehall II, glucose, HDL measures, and creatinine are among the frequently chosen predictors of incident dementia [11], which implied that the MIND diet might have favorable metabolomic profiles that could further enhance cognitive health.

**FIGURE 2:**
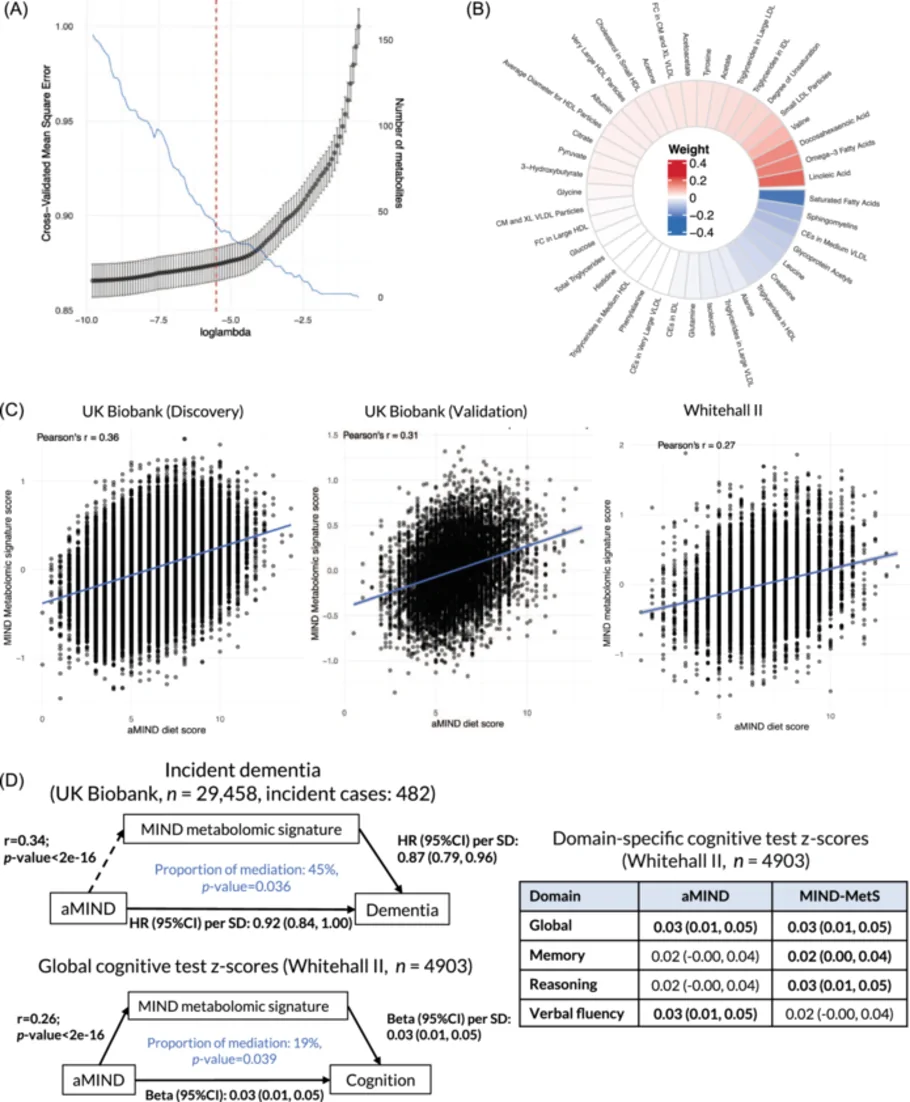
Construction of the MIND‐MetS and mediation analysis. (A) Feature selection process from the NMR metabolites in the elastic net for MIND‐MetS. (B) The weights of the metabolites selected in the MIND‐MetS. (C) The correlation of MIND‐MetS with the aMIND in each data set. (D) The associations of MIND‐MetS with cognitive outcomes. The proportion of mediation was calculated as the proportion of coefficient reduction for the aMIND after further adjusting the models for MIND‐MetS, and 95% CIs were generated using non‐parametric bootstrapping. CI, confidence interval; HR, hazard ratio; MIND, Mediterranean‐DASH Diet Intervention for Neurodegenerative Delay; MetS, metabolomic signature score; NMR, nuclear magnetic resonance; SD, standard deviation.

We applied elastic net model as dimension‐reduction approach, regressing the metabolites on the aMIND, to construct a metabolomic signature score (MIND‐MetS) for the MIND diet (Figure 2A). Among the 168 metabolites, 41 were selected for the MIND‐MetS construction (Table S5), with positive weights assigned to linoleic acid, total omega‐3 fatty acids, docosahexaenoic acid, etc., and negative weights to saturated fatty acids, sphingomyelins, cholesteryl esters in medium VLDL, glycoprotein acetyls, etc. (Figure 2B). The MIND‐MetS was significantly correlated with the aMIND (Pearson's *r* = 0.36 in the discovery set, 0.31 in the internal prospective validation cohort of UKB, and 0.27 in the external validation cohort of WHII, all *p* values < 0.001, Figure 2C). The moderate correlations might result from the inevitable dietary and metabolomic measurement errors [12], the complexity in the dietary pattern (rather than a specific nutrient or food group), and other factors affecting metabolite levels [8].

### MIND metabolomic signature and cognitive outcomes

We assessed the associations of the aMIND and MIND‐MetS with incident dementia in UKB (482 cases in 11.8 years' follow‐up) and cognitive function z‐scores in WHII participants who were aged 55 years or older at baseline. Both the aMIND and the MIND‐MetS were significantly associated with lower risk of dementia, with hazard ratios (HRs) per standard deviation (SD) increment being 0.92 (95% confidence intervals [CIs]: 0.84, 1.00, *p*‐value = 0.045) and 0.88 (0.79, 0.97, *p*‐value = 0.008), respectively (Figure 2D and Table S6). The association between the aMIND and incident dementia was mediated by the MIND‐MetS by 46% (*p*‐value for mediation = 0.038). In the WHII, each SD increment in the aMIND and the MIND‐MetS was associated with 0.03 (95% CI: 0.01, 0.05) and 0.03 (95% CI: 0.01, 0.05) higher cognitive test z‐scores, respectively. We observed similar associations in domain‐specific cognitive function. The association between the aMIND and cognitive function was partially mediated by the MIND‐MetS (proportion = 19%, *p*‐value for mediation = 0.039). The associations of the aMIND diet score and the MIND‐MetS with incident dementia and global cognitive function z‐score (Tables S7 and S8) were similar across most subgroups of participants defined by age, sex, education, ethnicity, BMI, and hypertension status. The associations also remained robust in a series of sensitivity analyses (Table S9).

Previous studies have shown the associations of the MIND diet with more favorable cognitive outcomes, including better cognitive function [6], slower cognitive decline [5], lower risk of dementia [7], and more favorable brain imaging markers [13]. Our research supports the potential of dietary interventions in mitigating the risk of dementia through metabolic pathways and may serve as a hint for future studies into the targets of dietary intervention for cognitive health. As NMR‐based metabolomic measurements are now widely accessible, they enable reproducible, accurate, and cost‐effective analysis of metabolic profiles [14, 15]. Our results underscore the importance of considering and monitoring metabolic health alongside dietary modifications when managing patients at risk of cognitive decline or neurodegenerative disorders. In public health practices, promoting the adoption of dietary patterns, like the MIND diet, that are not only beneficial for metabolic health but also associated with better cognitive outcomes, may have a substantial impact on population‐level brain health.

Our findings supported that the relation between the MIND diet and cognition is partially explained by metabolic and vascular health. Omega‐3 fatty acids, especially DHA, is important for central nervous system function [16], while several systemic inflammatory markers (such as glycoprotein acetyls [15]) and amyloid‐formation‐related amino acids (phenylalanine [17]) have been individually associated with higher dementia risk. For example, LDL‐C may promote neuroinflammatory responses and enhance the influence of Aβ deposition on tau pathology, a key factor in Alzheimer's disease (AD) progression, as well as inhibiting the activity of cathepsin D in autophagic lysosomes [18]. While the role of high‐density lipoprotein cholesterol (HDL‐C) in AD have not fully understood, it might support the synthesis of new membranes and potentially helping restore synaptic connections and inhibit the accumulation of cerebral amyloid angiopathy [19]. A large portion of the association remained unexplained, which was likely a mixed result of other biological pathways and errors in measuring diet, metabolites, and cognitive function. Further studies are needed to clarify and understand the underlying mechanisms.

Our study has several limitations. First, because this study is based on observational cohorts, our findings may not fully represent causal relations and await long‐term randomized controlled trials to provide causal evidence. The mediation analysis was based on a simplified model of the pathways through which diet may influence cognitive health, and future studies are needed to investigate other mediating factors. Secondly, the study populations in WHII and UKB were both from the UK and mostly consisted of White individuals. While they are two of the most representative and long‐term cohort studies in the UK, the generalizability of the findings to other populations warrants future examination. Additionally, the dietary assessments had certain levels of measurement errors (especially when the number of the 24‐h diet recalls is limited), thus diluting the potential associations and masking potential metabolites influenced by the diet. The single measurement may not fully capture the dynamic nature of an individual's metabolic status over time, especially given that multiple factors (such as statin use) may influence its accuracy in representing dietary intake. Furthermore, the cognitive assessments which may not capture all the cognitive domains and are also subject to measurement errors. Finally, the UKB and WHII may have a possible sample overlap, and the findings still need to be further validated. Nevertheless, the potential overlap might be trivial, considering the baseline age inclusion criteria in both cohorts (35−55 years in 1985−1988 for WHII and 37−73 years in 2006−2010 for UKB).

## 2 CONCLUSION

In two large population‐based studies of UK individuals, participants with better adherence to the MIND diet had higher levels of beneficial metabolites, including unsaturated fats and high‐density lipoprotein features and lower levels of potentially detrimental metabolites, such as glycoprotein acetyls and phenylalanine. The metabolomic signature of the MIND diet (MIND‐MetS) was independently associated with lower dementia risk and better global and domain‐specific cognitive function and partially mediated the associations between the MIND diet and cognitive outcomes. These findings support the potentially beneficial role of the MIND diet in cognitive health through metabolic health maintenance. Future investigations are required to further comprehend the underlying mechanisms and to assess the validity of the results in other populations.

## 3 METHODS

The inclusion flow chart of the study population is illustrated in Figure S1. The UKB used a validated tool for 24‐h diet recall in 2009–2012, and the WHII collected dietary data using a 127‐item food frequency questionnaire (FFQ) in 1991–1993 and 1997–1999. In both cohorts, we calculated a 15‐unit aMIND to reflect MIND diet adherence based on validated dietary questionnaires (Table S10) [20]. Nightingale [10, 14], a high‐throughput nuclear magnetic resonance (NMR) metabolomics platform, performed the metabolite assays.

The UKB identified dementia cases from linkage to electronic health records, including primary care, hospital admissions and the death registry, coded with the ICD‐10 and Read coding system from 2006 to 2010 until December 2022. In the WHII, global and domain‐specific cognitive function was measured using a cognitive test battery in 1997−1999, 2002−2004, 2007−2009, and 2012−2013. We used multivariable‐adjusted linear regression models to assess the associations of aMIND with metabolite z‐scores. We constructed a MIND‐MetS using elastic net regression to train the weights for all 168 metabolites in the UKB discovery cohort and assessed its association with incident dementia using Cox proportional hazard models and global and domain‐specific cognitive function z‐scores using linear mixed models. The comprehensive descriptions on the study population, measurements, and statistical analysis are available in the Supporting Information.

## AUTHOR CONTRIBUTIONS

**Hui Chen**: Conceptualization; methodology; writing—original draft; formal analysis; visualization. **Jie Shen**: Methodology; visualization; writing—review and editing. **Yang Tao**: Validation; visualization; writing—review and editing; methodology. **Yaodan Zhang**: Visualization; writing—review and editing. **Mengyan Gao**: Writing—review and editing. **Yuan Ma**: Writing—review and editing; validation; methodology; investigation. **Yan Zheng**: Writing—review and editing; methodology; validation; investigation. **Geng Zong**: Methodology; investigation; writing—review and editing; supervision; resources; data curation. **Qing Lin**: Writing—review and editing. **Lusha Tong**: Writing—review and editing. **Changzheng Yuan**: Funding acquisition; project administration; software; writing—review and editing; conceptualization; methodology; supervision.

## CONFLICT OF INTEREST STATEMENT

The authors declare no conflicts of interest.

## ETHICS STATEMENT

The UK Biobank was approved by the NorthWest Center for Research Ethics Committee (11/NW/0382), and the Whitehall II study was approved by the University College London Hospital Committee on the Ethics of Human Research (85/0938).

## Supporting information

The online version contains supplementary figures and tables available.

**Figure S1**: Participant inclusion flow chart.

**Table S1**: Characteristics of study participants.
**Table S2**: Missing rates of NMR metabolites in the study population.
**Table S3**: Coefficients for the associations between aMIND diet score with metabolites.
**Table S4**: Coefficients for the associations between MIND diet components with metabolites.
**Table S5**: Weights of metabolites for the MIND metabolomic signature score.
**Table S6**: Associations of the alternate MIND diet score and MIND metabolomic signature score (per standard deviation increment) with incident dementia in the UK Biobank (discovery and validation combined) and global cognitive function z‐scores in the Whitehall II study.
**Table S7**: Association of the MIND diet and its metabolic signature (per standard deviation) with risk of dementia in study subgroups.
**Table S8**: Association of the MIND diet and its metabolic signature (per standard deviation) with global cognitive function z‐score in study subgroups.
**Table S9**: Association of the MIND diet and its metabolic signature (per standard deviation) with risk of dementia and global cognitive function z‐score in sensitivity analysis.
**Table S10**: Scoring criteria for the alternate MIND diet score (aMIND).

## Data Availability

Data are available on request for bone fide investigators from managing institutions of the two studies: https://www.ucl.ac.uk/epidemiology-health-care/research/epidemiology-and-public-health/research/whitehall-ii for the Whitehall II study and https://www.ukbiobank.ac.uk for the UK Biobank. Software used in this study is publicly available. The code of the current study can be accessed at https://github.com/YuanLabZJU/mind-metab-sharing. Supplementary materials (methods, figures, tables, graphical abstract, slides, videos, Chinese translated version, and update materials) may be found in the online DOI or iMeta Science http://www.imeta.science/imetaomics/.
