# Supplementary material for: Circulating metabolomic profile of the MIND diet and its relation to cognition in middle‐aged and older adults
Source: IMetaOmics. 2025 Feb 10;2(1):e61. doi: 10.1002/imo2.61 (PMC12806510; doi:10.1002/imo2.61)
Supplement: Supplementary file 1 — Figure S1: Participant inclusion flow chart. [file IMO2-2-e61-s001.docx]

**Supporting information to**

**Circulating metabolomic profile of the MIND diet and its relation to cognition in middle-aged and older adults**

**Running title:** Circulating metabolomic profile of the MIND diet and cognition

Hui Chen^1^, Jie Shen^1^, Yang Tao^2^, Yaodan Zhang^1^, Mengyan Gao^1^, Yuan Ma^3^, Yan Zheng^4^, Geng Zong^5^, Qing Lin^6,7^, Lusha Tong^7^, Changzheng Yuan^1,8^*

^1^School of Public Health, the Second Affiliated Hospital, Zhejiang University School of Medicine, Hangzhou, 310058, China

^2^Cancer Epidemiology Unit, Nuffield Department of Population Health, Oxford University, Oxford, UK

^3^Department of Epidemiology, Harvard T. H. Chan School of Public Health, Boston, Massachusetts, 02445, USA

^4^State Key Laboratory of Genetic Engineering, Human Phenome Institute, and School of Life Sciences, Fudan University, Shanghai, China

^5^CAS Key Laboratory of Nutrition, Metabolism and Food Safety, Shanghai Institute of Nutrition and Health, University of Chinese Academy of Sciences, Chinese Academy of Sciences, Shanghai, China

^6^Department of Neurology, the First People’s Hospital of Taizhou, Taizhou, China

^7^Department of Neurology, the Second Affiliated Hospital, Zhejiang University School of Medicine, Hangzhou, 310058, China

^8^Department of Nutrition, Harvard T. H. Chan School of Public Health, Boston, Massachusetts, 02445, USA

*Correspondence: [chy478@zju.edu.cn](mailto:chy478@zju.edu.cn) (Changzheng Yuan)

**METHODS**

**Study population**

UK Biobank (UKB) is a large sample (~0.5 million) of the UK general population aged 37–73 years recruited from 22 centres between 2006 and 2010 [1]. Whitehall II (WHII) study is a prospective cohort study recruiting 10,308 individuals recruited from  Whitehall departments in London between 1985 and 1988 [2]. The inclusion flow chart of the study population was illustrated in Figure S1. Conceptually, we hypothesize that the metabolites were mediators of the associations of the diet-cognition association, so blood sample needed are collected concurrently or after with diet assessment. In the discovery phase of our study, we included UKB participants who were recruited after April 2009, when the touchscreen diet recall was introduced to the recruitment. The blood sample was also collected at recruitment. We then included participants who were recruited before April 2009, completed online diet assessment in 2011−2012, and completed blood sample collection at the 1^st^ follow-up in 2012−2013 [3] as an internal prospective validation cohort. To validate the findings in an external population, we included participants from the WHII study who completed diet assessments at Phase 3 (1991−1993), Phase 5 (1997−1999), or both, and finished blood sample collection at Phase 5. Specifically, we defined dietary assessment responses with unreasonably high (> 4500 kcal) or low (< 500 kcal) energy intakes as invalid dietary data and excluded them from analysis. To reduce reverse causation and mitigate measurement error in diet assessment, we excluded participants with prevalent dementia or incident dementia within the first 5 years of follow-up for analysis. In the analysis on the cognitive function and dementia, we further restricted the analysis to participants who aged ≥ 55 at baseline following a previous study [4]. Because early-onset and late-onset dementia can differ significantly with respect to pathophysiological changes, we further excluded incident dementia cases before the age of 65 from analysis (9 in UKB and 0 in WHII), defined as early-onset dementia.

The UKB study was approved by the NorthWest Centre for Research Ethics Committee (11/NW/0382) and the WHII study was approved by the University College London Hospital Committee on the Ethics of Human Research (85/0938). Written informed consent was obtained from all participants.

**Dietary assessment**

The UKB utilized the WebQ, a validated tool for 24-h diet recall, on five occasions between 2009 and 2012 [5,6], which asked participants the number of servings of each food group that they consumed yesterday[7]. The WHII collected dietary data using extensively validated food frequency questionnaires (FFQs) consisting of 127 items in 1991−1993 (Phase 3) and 1997−1999 (Phase 5) [8,9], where participants selected frequency of consumption of each food item in the past 12 months, and the options ranged from ‘never or < 1 times per month’ to ‘more than 3 per day’[10]. In this study, we slightly modified the original MIND diet score to use tertile-based rankings rather than the pre-defined cut-off points for each food group. We referred to this score as the alternate MIND diet score (aMIND), mimicking the previous strategy of assessing the adherence to a Mediterranean diet with an alternate Mediterranean diet score (aMed). This score would better capture the variation among participants on the intake of the pivotal food groups of the MIND diet among our populations. We categorized the food items into predefined food groups that are part of the MIND diet and calculated a 14-unit (in WHII) or 15-unit (in UKB) aMIND, as described in our previous study [11]. The healthy food groups included green-leafy vegetables, other vegetables, berries, nuts, beans, whole grains, non-fried fish, non-fried poultry, and olive oil use (not available in the WHII). We assigned a score of 1 for the highest tertiles of these food groups, 0.5 for the medium tertiles, and 0 for the lowest tertiles. Conversely, the unhealthy food groups, including cheese, butter and margarine, red meat and products, fast fried foods, pastries, and sweets, were assigned a score of 1 for the lowest tertiles, 0.5 for the medium tertiles, and 0 for the highest tertiles. For wine, which is recommended for moderate intake, we assigned a score of 1 to participants with moderate intake and 0.5 and 0 to those with over or non-consumption, respectively. We summed the scores from all food groups to obtain the aMIND, with a higher score indicating better adherence to the MIND diet. For participants who completed the dietary assessments multiple times, we calculated their average food group intake and used the average values to calculate the aMIND diet score. In a sensitivity, we have alternatively followed the scoring approach based on food frequency questionnaire (FFQ) proposed by Morris et al. in 2015 [12] in the Whitehall II study, which was calculated from pre-defined cut-offs rather than tertile-based rankings.

**NMR metabolomics**

Serum samples of WHII participants were collected and stored at -80°C in 1997−1999 and plasma samples of UKB participants in 2012−2013, as described in previous literature [13,14]. Both studies used the Nightingale platform (Helsinki, Finland) [15], a high-throughput nuclear magnetic resonance (NMR) metabolomics platform for metabolite assays. The WHII analyzed a total of 233 metabolic biomarkers (152 directly measured and 81 percentage/ratio measures), and the UKB NMR metabolomics data included a total of 249 metabolic biomarkers (168 directly measured and 81 ratio measures). To ensure consistency, we excluded derived percentage or ratio measures in both cohorts, and concentrations above the detection limit but below the quantification limit were set to zero. Outliers for each metabolite were also excluded from analysis, defined as values more than 9 SDs away from the mean [14]. The formal analysis included 152 and 168 metabolites in WII and UKB, respectively. The metabolites included lipoprotein subclasses (VLDL, IDL, LDL, and HDL), cholesterol-related metabolites, lipid-related metabolites, fatty acid-related metabolites, apolipoprotein-related metabolites, glycolysis-related metabolites, amino acids, ketone bodies, fluid balance metabolites, and inflammation-related metabolites. To cope with non-normality and outliers simultaneously, we used rank-based inverse normal transformation on the metabolite concentrations [16], and the outliers and skewed distributions would thus have minor influence on the study results.

**Cognitive function**

In this study, outcomes of interest included incident dementia in the UKB and cognitive test z-scores in the WHII. The UKB identified dementia cases from linkage to electronic health records (EHRs), including primary care, hospital admissions and the death registry, coded with the ICD-10 and Read coding system. The first two sources used International Classification of Diseases version 10 (ICD-10) for coding, and the primary care data are coded using the Read coding system (version 2 or 3) [17]. In the WHII, global and domain-specific cognitive function was measured using a cognitive test battery [18] at clinical examinations of Phases 5 (1997−1999), 7 (2002−2004), 9 (2007−2009), and 11 (2012−2013). Short term verbal memory was assessed with a 20-word free recall test. Alice Heim 4-I tests verbal and mathematical reasoning with a series of 65 items to identify patterns and infer principles and rules [19]. Participants were asked to write as many words beginning with “S” (phonemic fluency) and as many animal names (semantic fluency) as they could, forming a verbal fluency score [20]. We calculated the z-scores for the three domains using the mean and standard deviation (SD) at Phase 5 to allow for longitudinal analysis. The global cognitive score was calculated from the sum of the cognitive domain z-scores and re-standardized [18]. The mini-mental state examination, although also included in the WHII battery, was not used for current analysis because of its ceiling effects in the generally well-educated population.

**Covariates**

We collected information on sociodemographic and lifestyle factors from questionnaires prior to and concurrent with blood draw. The sociodemographic factors included age, sex, ethnicity (white or non-white), marriage and partnership status (in WHII), Townsend deprivation index (reflecting social deprivation, in UKB), highest level of formal education. Lifestyle factors included physical activity (low, medium, and high), smoking (current, former, or never). Alcohol intake was not considered a confounder because it is a component of the MIND diet. Body mass index was calculated from objectively measured weight and height in physical examination and categorized into under- or normal weight (< 25.0 kg/m^2^), overweight (25.0−< 30.0 kg/m^2^) and obesity (≥ 30.0 kg/m^2^). Health conditions included hypertension, diabetes, and cardiovascular diseases (myocardial infarction, stroke, and heart failure), all ascertained through self-reports, physical examinations, blood biochemical assays and linkage to the electronic health records (coded by International Classification of Diseases), as previously described [21]. Total energy intake was calculated concurrently with the aMIND, summing calories from each food item to represent the total energy intake of each individual using UK-based food composition table and database. The proportion of missing values was the highest for physical activity (19%) in the UKB, and BMI (13.3%) and physical activity (7.7%) in the Whitehall II study. All other covariates had a missing rate of < 2%. We imputed the missing values using multiple imputation by chained equations [22].

**Statistical analysis**

We described the participants’ characteristics at baseline (1997−1999 for WHII and 2009−2012 for UKB). Normally distributed variables were described using mean (SD) and categorical variables using number (percentage).

Firstly, we assessed the associations of aMIND (primarily) and the food groups in the MIND diet (secondary) with NMR-measured metabolite z-scores using linear regression with adjustments for age, sex, total energy intake, ethnicity, marriage and partnership status (in WHII), Townsend deprivation index (reflecting social deprivation, in UKB), years (WHII) or highest level (UKB) of formal education, physical activity (low, medium, and high), smoking (current, former, or never), BMI (under- or normal weight, overweight, and obesity), and history of hypertension, diabetes, and cardiovascular diseases. We used a per 3-unit increment in the aMIND as exposure variable because it approximates a 2-standard deviation increment and could represent a top-to-bottom tertile difference. When assessing the associations of the food groups in the MIND diet, we used top vs. bottom tertiles as the scale for coefficients, and moderate v. non-moderate intake was used for alcohol intake. We conducted Benjamini-Hochberg adjustment for *p*-values to account for false discovery rate (FDR). Metabolites showing significance after Benjamini-Hochberg adjustment with the same directions in all three cohorts were identified as consistent metabolites. As we only had statin use data in the UKB, we conducted a sensitivity analysis by adjusting for statin use when assessing the associations between the aMIND diet score and metabolites in the UKB discovery cohort.

Secondly, we constructed a MIND metabolomic signature score (MIND-MetS) using elastic net regression to train the weights for all 168 metabolites in the UKB discovery cohort. The penalty term in the elastic net regression model allows for flexible dimension reduction and coefficient shrinkage. We used ten-fold cross-validation to calculate the mean squared error reflecting the model fit and avoid overfitting. The weights trained from the discovery cohort were then carried to the internal and external validation cohort. In each cohort, we calculated the Pearson’s *r* to assess the correlation between the metabolic score and the aMIND calculated from the dietary assessments.

In the third step, we examined the associations of aMIND (per SD) and MIND-MetS (per SD) with incident dementia using Cox proportional hazard (CPH) models and global and domain-specific cognitive function z-scores using linear mixed models adjusted for age, sex, total energy intake, ethnicity, Townsend deprivation index (reflecting social deprivation), highest level of formal education, physical activity (low, medium, and high), smoking (current, former, or never), BMI (under- or normal weight, overweight, and obesity), and history of hypertension, diabetes, and cardiovascular diseases, as mentioned above. The mixed model incorporated baseline cognitive function score as a covariate and a random intercept for each individual to capture the difference in baseline cognitive status of individuals. The CPH model follows patterns as:

$$h\left( t, E, X \right)= h_{0}\left( t \right)*exp (\beta_{e}*E + \sum_{1}^{m} \beta_{i}*X_{i})$$

where $E$ stands for the exposure variable, $X$ stands for covariates, and $\beta_{e}$ is the coefficient of interest, which equals to $log(Hazard Ratio)$. Similarly, the LMM model follows patterns as:

$$Y\left( i,t \right)=\beta_{0,i}+ \beta_{e}*E+\beta_{t}*t+ \sum_{1}^{m} \beta_{i}*X_{i}$$

where $i$ stands for each individual, $t$ stands for the calendar time of cognitive assessment, $\beta_{e}$ is the random random intercept for each individual, and $\beta_{e}$ is the coefficient of interest.

We performed causal mediation analysis when the associations between the aMIND and outcome was significant, assuming that the association of the MIND diet with cognitive function was partially mediated by the MIND-MetS. The analyses were adjusted as in the first step. Briefly, we followed the Baron and Kenny’s [23] steps for mediation analysis:

- Step 1: We regressed of the exposure variable (aMIND) on the outcome variable (incident dementia or cognitive test z-score). The CPH or LMM model used is described as above.
- Step 2: If the coefficient of interest in Step 1 was statistically significant (*p*-value < 0.05), we regressed mediator (MIND-MetS) on the aMIND:

$$MIND\_MetS=\beta_{0}+ \beta_{aMIND}*\mathrm{aMIND}$$

- Step 3: If $\beta_{aMIND}$ in Step 2 was statistically significant (*p*-value < 0.05), we added the metabolomic signature score to Step 1 model, with the aMIND and all other covariates retained.

We then adopted the Preacher and Hayes bootstrapping method [24] (500 times) to calculate the corresponding confidence intervals and *p*-values for proportion of mediation in a non-parametric test.

We further performed subgroup analysis to test whether the associations of the aMIND and MIND-MetS with cognitive function differed by age, sex, education, ethnicity, BMI, and hypertension status. We tested for interaction using likelihood ratio test between models with and without the cross-product term of the exposure variable with the stratification variable. In the sensitivity analysis, we first excluded participants with diabetes or cardiovascular diseases because they may experience metabolic dysfunction. Secondly, we further adjusted the models for alcohol drinking behaviour (current, former, or never drinkers). We also tested for non-linearity by entering a quadratic term in the model and assessed its significance using analysis of variance.

Data analysis and visualization was conducted using SAS (Version 9.4) and R (Version 4.1.0) from January to April 2023 and modified and revised in January 2024. We used ‘lmer4’ package in mixed modelling and ‘mediation’ package in mediation analysis. *p*-values (FDR-adjusted) below 0.05 was considered indicators of statistical significance.

**References**

1. Bycroft, Clare, Colin Freeman, Desislava Petkova, Gavin Band, Lloyd T. Elliott, Kevin Sharp, Allan Motyer, et al. 2018. “The UK Biobank resource with deep phenotyping and genomic data.” *Nature* 562: 203-209. https://doi.org/10.1038/s41586-018-0579-z

2. Marmot, Michael G., Stephen Stansfeld, Chandra Patel, Fiona North, Jenny Head, Ian White, Eric Brunner, Amanda Feeney, George D. Smith. 1991. “Health inequalities among British civil servants: the Whitehall II study.” *The Lancet* 337: 1387-1393. https://doi.org/10.1016/0140-6736(91)93068-K

3. Carter, Jennifer L., Sarah Lewington, Carmen Piernas, Kathryn Bradbury, Timothy J. Key, Susan A. Jebb, Matthew Arnold, Derrick Bennett, Robert Clarke. 2019. “Reproducibility of dietary intakes of macronutrients, specific food groups, and dietary patterns in 211 050 adults in the UK Biobank study.” *Journal of Nutritional Science* 8: e34. https://doi.org/10.1017/jns.2019.31

4. Cornelis, Marilyn C., Puja Agarwal, Thomas M. Holland, Rob M. van Dam. 2023. “MIND dietary pattern and its association with cognition and incident dementia in the UK Biobank.” *Nutrients* 15: 32. https://www.mdpi.com/2072-6643/15/1/32

5. Liu, Bette, Heather Young, Francesca L. Crowe, Victoria S. Benson, Elizabeth A. Spencer, Timothy J. Key, Paul N. Appleby, Valerie Beral. 2011. “Development and evaluation of the Oxford WebQ, a low-cost, web-based method for assessment of previous 24 h dietary intakes in large-scale prospective studies.” *Public Health Nutrition* 14: 1998-2005. https://doi.org/10.1017/S1368980011000942

6. Galante, Julieta, Ligia Adamska, Alan Young, Heather Young, Thomas J. Littlejohns, John Gallacher, Naomi Allen. 2016. “The acceptability of repeat Internet-based hybrid diet assessment of previous 24-h dietary intake: administration of the Oxford WebQ in UK Biobank.” *British Journal of Nutrition* 115: 681-686. https://doi.org/10.1017/S0007114515004821

7. Greenwood, Darren C, Laura J Hardie, Gary S Frost, Nisreen A Alwan, Kathryn E Bradbury, Michelle Carter, Paul Elliott, et al. 2019. “Validation of the Oxford WebQ online 24-hour dietary questionnaire using biomarkers.” *American Journal of Epidemiology* 188: 1858-1867. https://doi.org/10.1093/aje/kwz165

8. Akbaraly, Tasnime N., Archana Singh-Manoux, Aline Dugravot, Eric J. Brunner, Mika Kivimäki, Séverine Sabia. 2019. “Association of midlife diet with subsequent risk for dementia.” *JAMA* 321: 957-968. https://doi.org/10.1001/jama.2019.1432

9. Al-Shaar, Laila, Changzheng Yuan, Bernard Rosner, Stefanie B Dean, Kerry L Ivey, Catherine M Clowry, Laura A Sampson, et al. 2020. “Reproducibility and validity of a semiquantitative food frequency questionnaire in men assessed by multiple methods.” *American Journal of Epidemiology* 190: 1122-1132. https://doi.org/10.1093/aje/kwaa280

10. Yue, Yiyang, Changzheng Yuan, Dong D. Wang, Molin Wang, Mingyang Song, Zhilei Shan, Frank Hu, Bernard Rosner, Stephanie A. Smith-Warner, Walter C. Willett. 2022. “Reproducibility and validity of diet quality scores derived from food-frequency questionnaires.” *The American Journal of Clinical Nutrition* 115: 843-853. https://doi.org/https://doi.org/10.1093/ajcn/nqab368

11. Chen, Hui, Michelle M. Dunk, Binghan Wang, Mengjia Zhao, Jie Shen, Geng Zong, Yuesong Pan, Lusha Tong, Weili Xu, Changzheng Yuan. 2024. “Associations of the Mediterranean-DASH Intervention for Neurodegenerative Delay diet with brain structural markers and their changes.” *Alzheimer’s & Dementia* 20: 1190-1200. https://doi.org/10.1002/alz.13521

12. Morris, Martha C., Christy C. Tangney, Yamin Wang, Frank M. Sacks, Lisa L. Barnes, David A. Bennett, Neelum T. Aggarwal. 2015. “MIND diet slows cognitive decline with aging.” *Alzheimer’s & Dementia* 11: 1015-1022. https://doi.org/10.1016/j.jalz.2015.04.011

13. Machado-Fragua, Marcos D., Benjamin Landré, Mathilde Chen, Aurore Fayosse, Aline Dugravot, Mika Kivimaki, Séverine Sabia, Archana Singh-Manoux. 2022. “Circulating serum metabolites as predictors of dementia: a machine learning approach in a 21-year follow-up of the Whitehall II cohort study.” *BMC Medicine* 20: 334. https://doi.org/10.1186/s12916-022-02519-6

14. Buergel, Thore, Jakob Steinfeldt, Greg Ruyoga, Maik Pietzner, Daniele Bizzarri, Dina Vojinovic, Julius Upmeier zu Belzen, et al. 2022. “Metabolomic profiles predict individual multidisease outcomes.” *Nature Medicine* 28: 2309-2320. https://doi.org/10.1038/s41591-022-01980-3

15. Julkunen, Heli, Anna Cichońska, Mika Tiainen, Harri Koskela, Kristian Nybo, Valtteri Mäkelä, Jussi Nokso-Koivisto, et al. 2023. “Atlas of plasma NMR biomarkers for health and disease in 118,461 individuals from the UK Biobank.” *Nature Communications* 14: 604. https://doi.org/10.1038/s41467-023-36231-7

16. McCaw, Zachary R., Jacqueline M. Lane, Richa Saxena, Susan Redline, Xihong Lin. 2020. “Operating characteristics of the rank-based inverse normal transformation for quantitative trait analysis in genome-wide association studies.” *Biometrics* 76: 1262-1272. https://doi.org/https://doi.org/10.1111/biom.13214

17. Wilkinson, Tim, Christian Schnier, Kathryn Bush, Kristiina Rannikmäe, David E. Henshall, Chris Lerpiniere, Naomi E. Allen, et al. 2019. “Identifying dementia outcomes in UK Biobank: a validation study of primary care, hospital admissions and mortality data.” *European Journal of Epidemiology* 34: 557-565. https://doi.org/10.1007/s10654-019-00499-1

18. Singh-Manoux, Archana, Mika Kivimaki, M Maria Glymour, Alexis Elbaz, Claudine Berr, Klaus P Ebmeier, Jane E Ferrie, Aline Dugravot. 2012. “Timing of onset of cognitive decline: results from Whitehall II prospective cohort study.” *BMJ* 344: d7622. https://doi.org/10.1136/bmj.d7622

19. Heim, A.W. 1970. A.H.4 Group Test of General Intelligence Manual. *NFER Publishing Company Limited*, https://books.google.com/books?id=beYqHQAACAAJ

20. Borkowski, John G., Arthur L. Benton, Otfried Spreen. 1967. “Word fluency and brain damage.” *Neuropsychologia* 5: 135-140. https://doi.org/https://doi.org/10.1016/0028-3932(67)90015-2

21. Chen, Hui, Klodian Dhana, Yuhui Huang, Liyan Huang, Yang Tao, Xxiaoran Liu, Debora Melo van Lent, et al. 2023. “Association of the Mediterranean Dietary Approaches to Stop Hypertension Intervention for Neurodegenerative Delay (MIND) Diet with the risk of dementia.” *JAMA Psychiatry* 80: 630-638. https://doi.org/10.1001/jamapsychiatry.2023.0800

22. White, Ian R., Patrick Royston, Angela M. Wood. 2011. “Multiple imputation using chained equations: Issues and guidance for practice.” *Statistics in Medicine* 30: 377-399. https://doi.org/https://doi.org/10.1002/sim.4067

23. Baron, Reuben M., David A. Kenny. 1986. “The moderator-mediator variable distinction in social psychological research: conceptual, strategic, and statistical considerations.” *Journal of Personality and Social Psychology* 51: 1173-1182. https://doi.org/10.1037//0022-3514.51.6.1173

24. Hayes, Andrew F., Kristopher J. Preacher. 2014. “Statistical mediation analysis with a multicategorical independent variable.” *British Journal of Mathematical and Statistical Psychology* 67: 451-470. https://doi.org/https://doi.org/10.1111/bmsp.12028


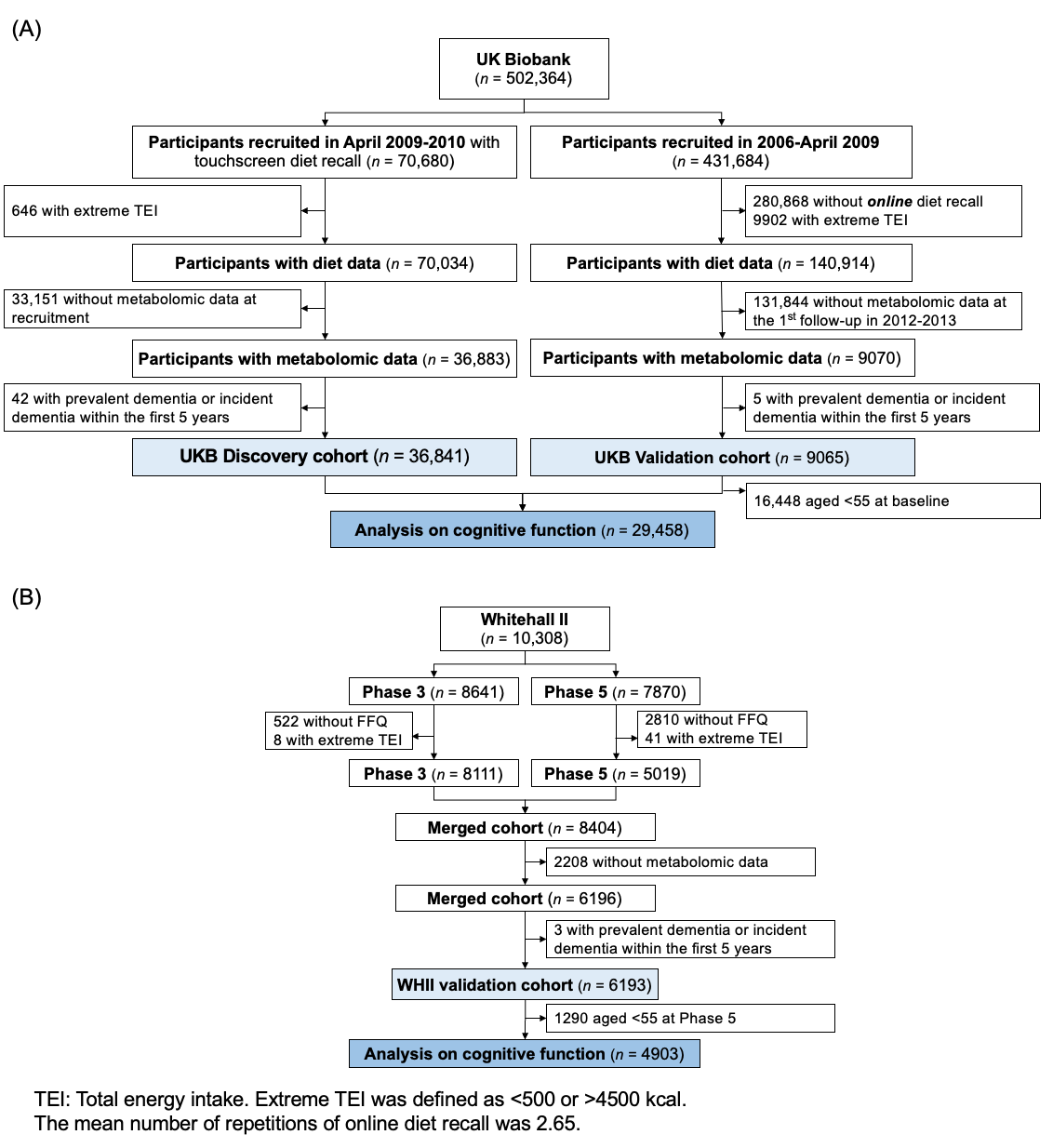


**Figure S1 Participant inclusion flow chart.**
